# Spark Plasma Diffusion Bonding of TiAl/Ti_2_AlNb with Ti as Interlayer

**DOI:** 10.3390/ma13153300

**Published:** 2020-07-24

**Authors:** Boxian Zhang, Chunhuan Chen, Jianchao He, Jinbao Hou, Lu Chai, Yanlong Lv

**Affiliations:** 1 School of Materials Science and Engineering, Dalian Jiaotong University, Dalian 116028, China; zhangbx0225@163.com; 2 Institute of Special Environments Physical Sciences, Harbin Institute of Technology, Shenzhen 518055, China; 3 Aeronautical Key Laboratory for Welding and Joining Technologies, AVIC Manufacturing Technology of Institute, Beijing 100024, China; houjinbao168@sina.com (J.H.); chailu408446026@163.com (L.C.); lvyanlong0210@126.com (Y.L.)

**Keywords:** spark plasma diffusion bonding, TiAl, Ti_2_AlNb, microstructure, elemental migration, microhardness, tensile properties

## Abstract

To solve the problem of poor weldability between TiAl-based and Ti_2_AlNb-based alloys, spark plasma diffusion bonding was employed to join a TiAl alloy and a Ti_2_AlNb alloy with a pure Ti foil as interlayer at 950 °C/10 KN/60 min. After welding, slow cooling was carried out at a rate of 5 °C/min, followed by homogenization at 800 °C for 24 h. The microstructural evolution and elemental migration of the joint were analyzed via a scanning electron microscope equipped with an energy dispersive spectrometer, while the mechanical properties of the joint were assessed via microhardness and tensile tests. The results show that the spark plasma diffusion bonding formed a joint of TiAl/Ti/Ti_2_AlNb without microcracks or microvoids, while also effectively protecting the base metal. Before heat treatment, the maximum hardness value (401 HV) appeared at the Ti_2_AlNb/Ti interface, while the minimum hardness value (281 HV) occurred in the TiAl base metal. The tensile strength of the heat-treated joint at room temperature was measured to be up to 454 MPa, with a brittle fracture occurring in the interlayer. The tensile strength of the joint at 650 °C was measured to be up to 538 MPa, with intergranular cracks occurring in the TiAl base metal.

## 1. Introduction

With the development of modern aerospace industry, it has become increasingly difficult for traditional materials to meet the requirements of aerospace structural components, that is, to be lightweight and to offer high performance simultaneously. Titanium aluminum alloys and bi-metal structures are getting increased attention as optimal solutions for this problem. Due to low density, high melting point, high specific modulus, and excellent creep and oxidation resistance, TiAl alloy is one of the important candidate materials to replace some Ni-base superalloys in the temperature range of 650–900 °C with the goal of weight loss [1,2]. Ti_2_AlNb alloy is a new type of lightweight high-temperature structural material developed after TiAl and Ti_3_Al alloys, with a high performance of fracture toughness and creep resistance caused by the addition of the Nb element [3,4]. For its good low-cycle-fatigue property and high creep resistance, Ti_2_AlNb alloy has been applied extensively in the aerospace field within 600–750 °C. Researching the welding of TiAl and Ti_2_AlNb is of significant importance, as this will allow the thrust-to-weight ratio of aerospace components to be improved and the application possibilities of such alloys to be broadened.

To date, fusion welding techniques such as laser welding [5,6,7,8] and electron beam welding [9,10,11,12] have been employed when joining TiAl-based alloys. It is found that defects such as high stress, hot cracks, and brittle intermetallic compounds often occur due to excessively fast cooling speed when laser welding and electron beam welding are used. Solid phase bonding is expected to be used for the achievement of high-quality bonding of TiAl alloy. Methods of solid phase welding, including linear friction welding [13] and conventional diffusion bonding [14,15], can generate recrystallized grains at the bonding interface of TiAl/Ti_2_AlNb and block the crossing of cracks, thus further improving the welding quality.

Spark plasma diffusion bonding (SPDB) is a rapid-bonding method that introduces the characteristics of pulse current to solid phase diffusion bonding, promoting atomic diffusion and material plastic deformation. The key advantage to this method is that the heat is mainly generated at the interface, which is definitely different from the overall heating of the traditional hot-press diffusion bonding. SPDB has been applied to the direct diffusion bonding of Ti-48Al-2Cr-2Nb alloy [16], Ti-45Al-7Nb-0.3W (at.%) alloy [17], TZM30 alloy, WRE alloy [18], and HS-6-7-6-10-0.1LaB6 powder metallurgy high speed steel (PMHSS) [19] and to the connection of SiC [20] with Ta-5W as the interlayer.

When metallurgical factors are taken into consideration, it is of great importance to select the appropriate interlayer to obtain high-quality welded joints. In the diffusion bonding of TiAl alloys, there are many kinds of interlayers. Ti-Nb [21], Ti-Ni-Nb [22] and TiZrCuNi [23] have all been used to improve the performance of a TiAl alloy bonding interface. It has been found that Ti has good compatibility with Ti_2_AlNb alloy and can inhibit the formation of brittle compounds.

Therefore, in order to obtain high-quality welded joints, pure Ti was selected as the interlayer in the SPDB of TiAl and Ti_2_AlNb. The microstructure and mechanical properties of the welded joints were then analyzed.

## 2. Experimental Procedure

**Figure 1:**
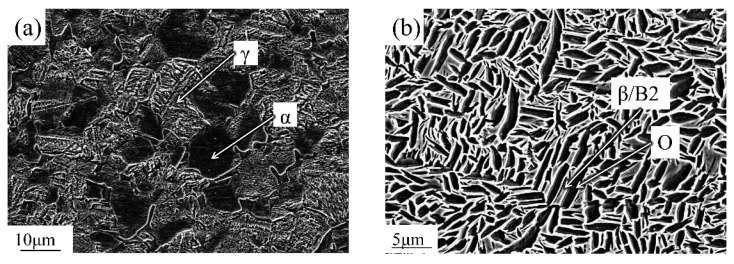
Microstructures of alloys (**a**) TiAl and (**b**) Ti_2_AlNb.

An as-extruded TiAl intermetallic compound with a nominal composition of Ti-46Al-2Cr (at.%) made by Institute of Metal Research, Chinese Academy of Sciences (Shenyang, China) was selected. The room-temperature microstructure of this material is shown in Figure 1a. The material’s nearly fully lamellar structure consisted of α phases and an alternate lamellar of γ phases. Forged Ti_2_AlNb alloy with nominal composition of Ti-22Al-27Nb (at.%) made by Central Iron & Steel Research Institute (Beijing, China) was also selected. Figure 1b shows the base metal of this material after the 800 °C annealing. The resulting microstructure was composed of massive O phases that were distributed on the β/B_2_ matrix. As one can see from the image, the O phase is dark and looks gray, while the B_2_ phase is bright white. Pure titanium TA1 with 0.1 mm thickness was selected as the interlayer. The base metal to be bonded was machined into a rod with a diameter of 40 mm and a length of 60 mm.

**Figure 2:**
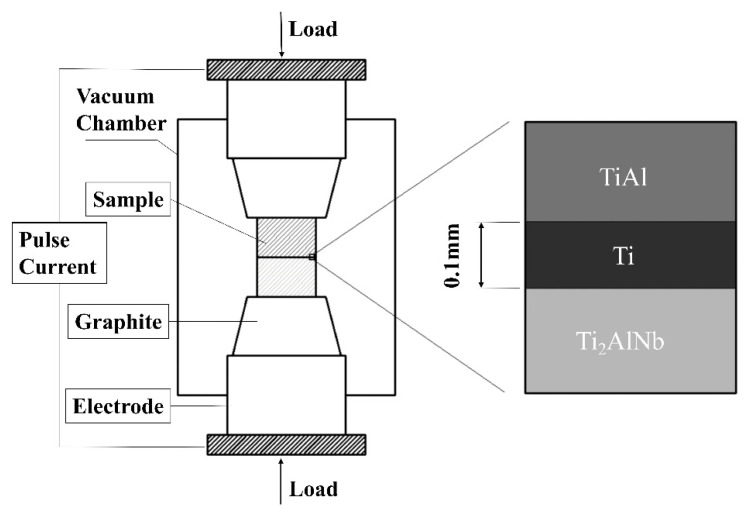
Schematic diagram of the spark plasma diffusion bonding method.

The diffusion welding test was carried out using HPD-25-HV/SP spark plasma diffusion equipment manufactured by FCT Systeme GmbH (Rauenstein, Germany) with a temperature of 950 °C, heating rate of 100 °C/min, pulse current of 7000 A, frequency of 1/9 ratio, vacuum degree of 2.3 × 10^−3^ Pa, pressure of 10 kN, and holding time of 60 min. Slow cooling was performed at a rate of 5 °C/min after welding. The welding assembly diagram is shown in Figure 2.

After welding, homogenizing heat treatment was performed using a vacuum heat treatment furnace manufactured by Centorr Vacuum Industries (Nashua, NH, USA) at 800 °C for 24 h. Microstructure specimens were obtained along the direction perpendicular to the welding surface, ground and polished, and corroded by aqueous solutions of hydrofluoric acid and nitric acid. The joint microstructure was observed and analyzed on a Zeiss Suppera55 scanning electron microscope at the test center of the China Aviation Manufacturing Technology Research Institute (Beijing, China), and the chemical composition of each phase produced by the interfacial reaction was analyzed by an Oxford Xmax energy dispersive spectrometer manufactured by Carl Zeiss (Shanghai, China). The hardness distribution of the joint was tested by an HXD-1000 microhardness tester manufactured by Shanghai Optical Instrument Factory (Shanghai, China) with a test load of 300 gf (294.2 N) and a holding time of 15 s. For the microhardness test, at least five points were selected for each of the following areas: TiAl base metal, TiAl/Ti interface, interlayer, Ti/Ti_2_AlNb interface, and Ti_2_AlNb base metal. After the heat treatment, the specimens were tested by a Z100 universal testing machine manufactured by ZwickRoell (Shanghai, China). for tensile tests at room temperature and high temperature. The tensile specimens were prepared according to the HB5214-96 standard.

## 3. Results and Discussion

### 3.1. Microstructural Evolution and Elemental Migration

**Figure 3:**
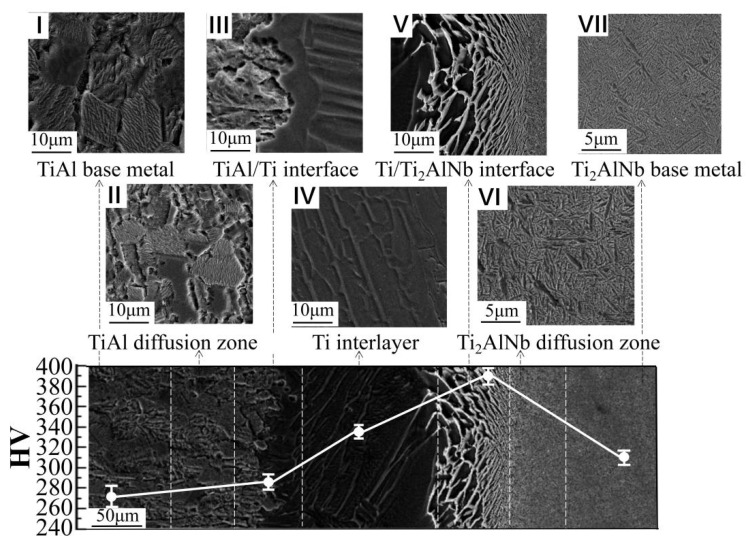
Microstructure of the TiAl/Ti_2_AlNb joint diffusion bonded with Ti foil at 950 °C for 60 min under pressure of 10 MPa and the Vickers microhardness profile across the joint.

The microstructures of the as-welded joints are shown in Figure 3. No welding defects were observed at the as-welded bonding interface of TiAl and Ti_2_AlNb in terms of microholes, microcracks, poor bonding, or other anomalies. Furthermore, a trend of microstructural variation was noticed from TiAl to Ti_2_AlNb. The width of the bonding interface was estimated to be 150 μm. To simplify the description of the results, the joint was divided into seven zones: the TiAl base metal (zone I), the diffusion zone of TiAl (zone II), TiAl/Ti interface (zone III), the Ti interlayer (zone IV), the Ti/Ti_2_AlNb interface (zone V), the diffusion zone of Ti/Ti_2_AlNb (zone VI), and the Ti_2_AlNb base metal (zone VII).

When using SPDB, the heat-affected range is quite narrow; hence, the TiAl and Ti_2_AlNb base metals were protected effectively. The analyses suggested very small cluster sizes of the α phase and the γ phase at the TiAl base metal in zone I. Besides, no significant changes in microstructure between zone I and zone II were recorded, indicating that welding temperature had little effect on the TiAl base metal. Zone VI represented the diffusion zone of Ti_2_AlNb, with a microstructure consisting of numerous O phases distributed in the B2 matrix. As expected, the growth of the O phase was noticed. The welding thermal cycle showed little influence on the Ti_2_AlNb base metal, leading to insignificant changes in the microstructure of zone VII.

Using pure Ti foil as an interlayer effectively improved the continuity in the microstructure of the joint. Zone III represents the transition region at which the TiAl base metal and Ti interlayer were bonded. Though the interface looked obvious, the bonding line appeared discontinuous and curved, consistent with intermetallic compound (IMC) Ti_3_Al based on the Ti/Al binary phase diagram [24]. Close to the bonding line, the microstructure of the TiAl base metal remained almost unchanged, while the interlayer side contained a coarse feathery microstructure. The formation of such a microstructure would most likely relate to interdiffusion between TiAl and Ti.

Zone IV comprised the middle area of the interlayer and was mainly composed of residual phases formed after diffusion and metallurgical reaction. Furthermore, the microstructure contained precipitated α phases, differently from that of the TiAl/Ti transition zone. Zone V represented the transition region of Ti/Ti_2_AlNb, where Ti and Ti_2_AlNb became diffusion bonded. No evident bonding interface was observed under the as-welded state of this transition zone, and the grain size looked finer than that of the TiAl/Ti interface (zone III). According to previous studies [25], such a microstructure would be more likely to contain α-Ti and β-Ti. These results were similar to those reported by Wang Ying [26], who determined that the dark grey acicular precipitates in the diffusion region were Nb-deficient and the light grey matrix phase was Nb-enriched. However, the dark grey acicular precipitates contained higher Al content than the light grey matrix phase. Thus, analysis of each phase in Ti_2_AlNb and Ti suggests that large amounts of Nb can form an infinite solid solution with β-Ti diffusion from Ti_2_AlNb to Ti. This led to the formation of a Nb-enriched zone near the interface, determined as a B2-enriched region.

**Figure 4:**
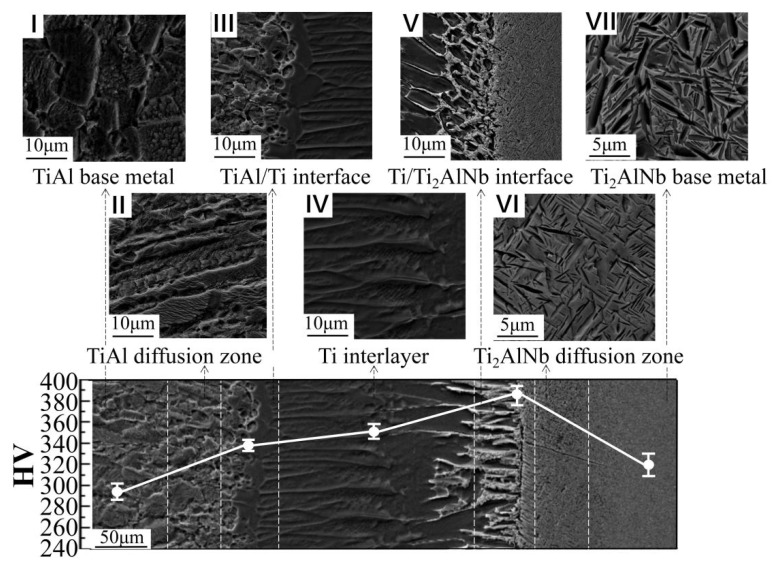
Microstructure of the TiAl/Ti_2_AlNb joint diffusion bonded with Ti foil after homogenizing heat treatment at 800 °C for 24 h and the Vickers microhardness profile across the joint.

Figure 4 shows the microstructure after homogenization by heat treatment. The microstructure of the TiAl base metal had no sharp changes; however, the phase transition occurred on the Ti_2_AlNb base metal. Zones I and III presented no obvious change after heat treatment. In zone II, Al further diffused to the interlayer, and more Ti was simultaneously replenished from the interlayer. This led to the fast growth and formation of the α phase along the diffusion direction. As a result, the fully lamellar microstructure obtained developed into laths following one direction. Zone VI and zone VII displayed no other differences except in terms of grain coarsening of the O phase, which led to sharpened phase boundaries of the O phase when compared to the as-welded joints. The effect of heat treatment on base-metal Ti_2_AlNb looked obvious as well. In particular, the B2 phase mostly transformed into the O phase and into small amounts of α2 due to the existence of some unevenly distributed Nb element, and transformation of B2→α2 only occurred in the Nb-less regions.

After homogenization, the most significant change appeared in zone V. Importantly, an interface emerged between Ti and Ti_2_AlNb with great differences in the microstructures of both sides near the interface. In the bulk of the interlayer, acicular β clusters were grown and arranged alternately on the α matrix, leading to river-like patterns that were perpendicular to the interface. At the Ti_2_AlNb side, fine needle-like structures appeared and arranged unorderly, similar to base-metal Ti_2_AlNb. During the slow cooling stage following heat treatment, both α2 and O phases partially precipitated again and coexisted with the β phase near the interface.

**Figure 5:**
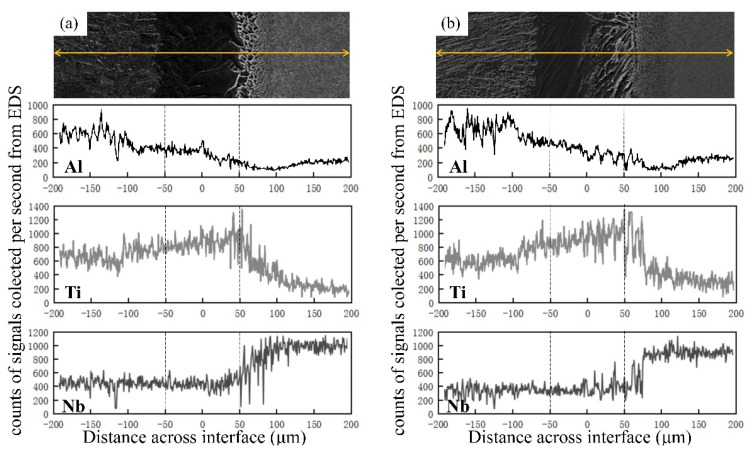
The elemental distributions along TiAl/Ti_2_AlNb alloy joints bonded with the Ti interlayer: (**a**) as-welded and (**b**) heat-treated.

The composition of the diffusion bonding interface was characterized by EDS, the results of which are provided in Figure 5. The extended width of the interlayer in the as-welded joint was estimated to be about 250 μm. This value was much higher than the thickness of the original interlayer, confirming the occurrence of ample interdiffusion between the interlayer and the base metals during the welding process.

Al content decreased gradually from the TiAl to the Ti_2_AlNb side; it dropped sharply in the Ti/Ti_2_AlNb transition zone, where the Al content reached the lowest level when compared to both base metals. By comparison, Ti content first increased and then declined from TiAl side to Ti_2_AlNb side, reaching its maximum value in the TiAl/Ti transition zone.

Ti and Al in the Ti_2_AlNb base metal diffused to the Ti interlayer more quickly than Nb in the base metal due to the smaller atomic radius of Ti and Al when compared to that of Nb. This led to enrichment of the transition zone of Ti_2_AlNb/Ti by Nb. The microstructural analyses revealed that only small amounts of finer O had precipitated from the B2 matrix in this region, indicating depletion of Al due to the region’s stable α-phase. These data were consistent with those from EDS.

The contents of Ti in both TiAl (Ti-46Al-2Cr) and Ti_2_AlNb (Ti-22Al-27Nb) were close to each other before welding, while Ti distribution varied significantly after welding. The Ti content in TiAl rose to that of the interlayer, while Ti content in Ti2AlNb remained almost unchanged. The distribution of Ti was related to the difference in diffusion rates of Al and Nb. The Al content was twice as high in TiAl than in Ti_2_AlNb, leading to an elevated concentration gradient of Al at the TiAl/Ti interface when compared to the Ti/Ti_2_AlNb interface.

Compared to Ti migrating from the interlayer, more Al located at the TiAl side migrated toward the interlayer. However, the amount of transferable Al at the Ti_2_AlNb side was nearly half that of TiAl. Coupled with the low diffusion rate of Nb, migration of Ti from interlayer to Ti_2_AlNb became more challenging, leading to an elevated concentration gradient of Ti near the Ti/Ti_2_AlNb interface.

After heat treatment, no obvious changes in the shape of the elemental distribution curves were observed, with the exception of the broadening of the interlayer width. Thus, the elements underwent further diffusion, but distribution regularities of Ti, Al, and Nb remained unchanged.

### 3.2. Microhardness Distribution

The microhardness distributions of the as-welded and heat-treated joints are displayed in Figure 3 and Figure 4. For the as-welded joints, the highest hardness appeared at the interface of Ti/Ti_2_AlNb, while the lowest was located at the TiAl base metal. Moreover, the heat-treated joints showed the same hardness distribution regularities, although certain changes occurred in different zones affected by heat treatment. The hardness remained almost stable in the interlayer, while it increased in the regions surrounding TiAl and decreased in the areas surrounding Ti_2_AlNb.

Al, as an α stabilization element that could diffuse from base metal to the interlayer during welding, promoted the transformation of some γ into α near the TiAl/Ti interface. This, in turn, increased the hardness at the TiAl/Ti interface between the TiAl base metal and the interlayer. The presence of more Nb at the Ti_2_AlNb/Ti interface led to the formation of substantial B2 phases in this area, further increasing the hardness of Ti_2_AlNb. On the other hand, the immigrated Al from TiAl provided an additional hardening effect by forming a substitutional solid solution [27].

To yield uniform distribution of α grains, the increase in hardness at the TiAl/Ti interface after heat treatment was associated with more diffusion of alloying Nb element after heat treatment in the region surrounding the α + γ phase in TiAl. This strengthened the TiAl solid solution and enhanced Vickers hardness. The importance of solid solution strengthening in Vickers hardness has been reported in previous literature [28]. At higher temperatures, the B2 phase decomposed into α2 and O, in which both plasticity and toughness of the α2 and O phases were better than those of the B2 phase. As a result, the hardness of the Ti/Ti_2_AlNb interface decreased.

### 3.3. Tensile Properties

**Figure 6:**
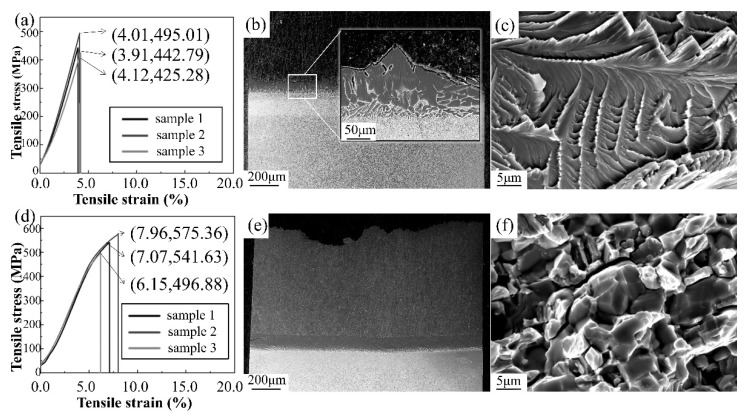
Tensile test at room temperature: (**a**) stress–strain curve, (**b**) location of fracture, (**c**) microfracture morphologies. Tensile test at 650 °C: (**d**) stress–strain curve, (**e**) location of fracture, (**f**) microfracture morphologies.

Since as-welded joints have no direct practical applications, only heat-treated joints were selected for tensile testing. The average tensile strengths were estimated to be 454 ± 41 MPa at room temperature and 538 ± 42 MPa at 650 °C. The stress–strain curve data are displayed in Figure 6a,d.

During tensile testing at room temperature, taking into account the very brittle nature of the IMC Ti_3_Al at room temperature [29], the higher Al content in TiAl led to more Ti_3_Al at the interface of TiAl/Ti when compared to Ti/Ti_2_AlNb. Furthermore, most joints fractured from the thin IMC layer precipitated at the TiAl/Ti interface, and a few others fractured at the Ti/Ti_2_AlNb interface, as shown in Figure 6b. At high temperature (650 °C), however, both interfaces were no longer the weakest parts of the whole joint, and the failure position moved to the TiAl base metal, as displayed in Figure 6e. The reason for this had to do with the increase in Ti_3_Al toughness and decline in brittleness at high temperatures. Moreover, another possible reason could be that the immigrated Nb markedly strengthened Ti_3_Al and the surrounding microstructures [30].

The micromorphology of the fracture is presented in Figure 6c,f. Both joints demonstrated brittle characteristics related to differences in the failure location of each joint, with transcrystalline fracture at room temperature and intercrystalline fracture at 650 °C. As described above, the IMC Ti_3_Al looked brittle at room temperature with a transgranular propagation of cracks. Numerous Ti_3_Al IMCs migrated to grain boundary and aggregated at high temperatures [31], leading to intercrystalline fracture mode.

## 4. Conclusions

Spark plasma diffusion bonding (SPDB) of TiAl alloy to Ti_2_AlNb alloy was achieved using a pure titanium interlayer. The microstructures and mechanical properties of the joints were investigated, and the major conclusions that can be drawn from this study are the following:

1. After welding, the joint of TiAl/Ti/Ti_2_AlNb is composed of TiAl; α + γ, α, α + β, and B2-rich duplex microstructures; and Ti_2_AlNb. The thickness of the interlayer was found to increase after homogeneous heat treatment due to the further diffusion of the elements Ti, Nb, and Al. This process resulted in the joint being composed of TiAl; lamellar α, α, α + β, and β + α2 + O phases; and Ti_2_AlNb.
2. The maximum hardness after welding (401 HV) appeared at the Ti_2_AlNb/Ti interface, while the minimum hardness (281 HV) occurred in the TiAl base metal. After heat treatment, the microhardness distribution at the joint became more uniform; it increased significantly from 309 HV up to 337 HV at the TiAl/Ti interface, while it decreased slightly at the Ti/Ti_2_AlNb interface.
3. At room temperature, the tensile fracture of the heat-treated joint occurred at the interlayer with an average tensile strength of 454 MPa, and it was found to be a transgranular fracture. On the other hand, at 650 °C, the fracture position moved to the TiAl base metal, with a tensile strength of 538 MPa, and was observed to be an intercrystalline fracture.
